# Solution Thermodynamics of l-Glutamic Acid Polymorphs from Finite-Sized Molecular Dynamics Simulations

**DOI:** 10.1021/acs.iecr.4c02558

**Published:** 2025-01-07

**Authors:** Fabienne Bachtiger, Aliff Rahimee, Lunna Li, Matteo Salvalaglio

**Affiliations:** Thomas Young Centre and Department of Chemical Engineering, University College London, London WC1E 7JE, U.K.

## Abstract

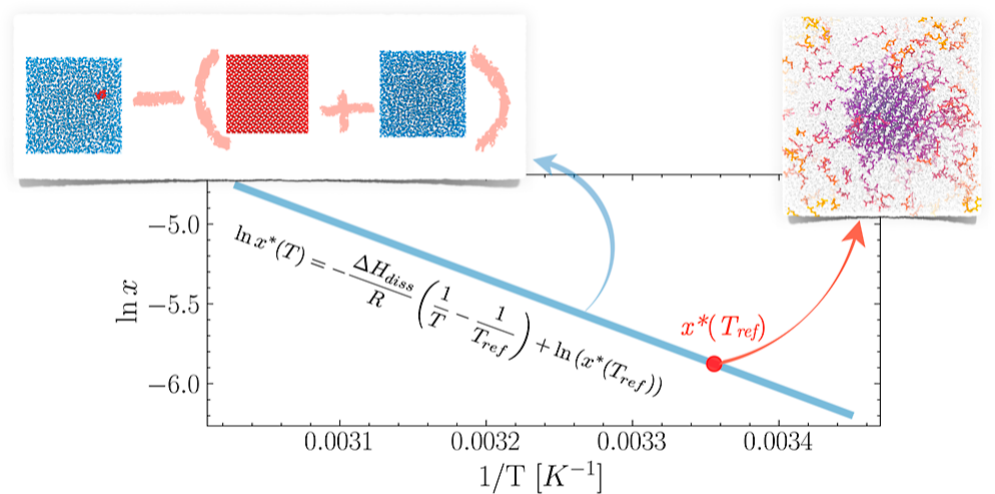

Efficiently obtaining atomic-scale thermodynamic parameters characterizing crystallization from solution is key to developing the modeling strategies needed in the quest for digital design strategies for industrial crystallization processes. Based on the thermodynamics of crystal nucleation in confined solutions, we develop a simulation framework to efficiently estimate the solubility and surface tension of organic crystals in solution from a few unbiased molecular dynamics simulations at a reference temperature. We then show that such a result can be extended with minimal computational overhead to capture the solubility curve. This enables an efficient and self-consistent estimate of the solubility and limit of solution stability associated with crystal nucleation in molecular systems from equilibrium molecular dynamics without the need for sophisticated free energy calculations. We apply our analysis to investigate the relative thermodynamic stability and aqueous solubility of the α and β polymorphs of l-glutamic acid. Our analysis enables an efficient appraisal of emergent ensemble properties associated with the thermodynamics of nucleation from solutions against experimental data, demonstrating that while the absolute solubility is still far from being quantitatively captured by an off-the-shelf point charge transferable force field, the relative polymorphic stability and solubility obtained from finite temperature simulation are consistent with the experimentally available information on glutamic acid. We foresee the ability to efficiently obtain solubility information from a limited number of computational experiments as a critical component of high-throughput polymorph screenings.

## Introduction

Molecular simulations of crystal nucleation and growth are key to understanding fundamental mechanisms and predicting the complex interplay of thermodynamic and kinetic factors that determine polymorphic outcomes and the emergence of growth shapes, ultimately enabling the development of digital design strategies for crystallization processes.^1−3^

The length and time scales of physics-based, atomistic simulations able to display the emergent, ensemble behavior of large numbers of molecules resulting in crystal growth typically require the use of semiempirical molecular models, usually based on classical force fields.^1,2,4^ Such models are often developed, trained, and benchmarked over *static* energy metrics to reproduce the equilibrium structure and energy of isolated molecules. Sometimes, ensemble properties of pure fluid phases are used in the validation and refinement of force fields;^5^ very rarely, force fields are parametrized and tested against ensemble properties of multicomponent systems. This is often due to the high computational cost and the subtle algorithmic complexities typical of the free energy methods used to obtain estimates of polymorph-specific solubility and relative polymorphic stability of *molecular models of* solutes and solvents.^2,6^ The complexity and computational cost of free energy methods also limit their applicability to systematic estimates of the temperature-dependent stability of crystal polymorphs, which are typically estimated employing approximate methods.^7−9^ As a result, solubility curves associated with *molecular models* of crystalline materials in explicit solvent are a rare find in the current literature^10,11^ and are often confined to benchmark systems or contributions focused on method development.

The lack of efficient methods for benchmarking equilibrium properties in multicomponent systems where solids and liquids coexist hampers the applicability of molecular simulations in quantitatively predictive studies where atomistic information is passed up the scales and informs simulations at the *meso* and *macro* scale.^12−16^

In this work, building on a detailed understanding of the finite-size thermodynamics of crystal-solution equilibria in finite-size isolated ensembles, we propose a systematic approach to provide a comprehensive overview of the collective, emergent properties of molecular solutes undergoing crystallization. Expanding on a previous contribution focused on the thermodynamics of liquid–liquid phase separations in systems of disordered peptides,^17^ we use a limited number of equilibrium molecular dynamics (MD) simulations (in this case 8–10) to efficiently estimate polymorph-specific solubility curves, relative stability, and limit of solution stability of l-glutamic acid (LGA) polymorphs.

LGA is an organic molecule that crystallizes in two polymorphic forms: α, metastable, and β, thermodynamically stable, across the entire range of temperatures experimentally accessible in aqueous solutions.^18−20^ The precipitation of α and β polymorphs from aqueous solutions is also well characterized experimentally: α LGA precipitates with faster nucleation kinetics, while β LGA appears due to a dissolution-mediated polymorphic transformation.^19−21^ The differences in the homogeneous nucleation rate of α and β,^22,23^ as well as the in-depth understanding of its crystallization mechanism, render LGA an ideal model compound to investigate polymorphic control strategies at the process level^24^ and to develop novel characterization techniques.^25^

In the following, we discuss the theoretical background, methods, and results obtained by analyzing the emergent behavior of MD simulations to obtain quantitative information about the thermodynamics associated with LGA nucleation from an aqueous solution. From a molecular modeling perspective, the wealth of experimental knowledge of LGA polymorphic crystallization makes it an ideal playground for testing new methods and critically assessing the strengths and weaknesses of the adopted molecular model against an experimental benchmark.

### Theoretical Background

**Figure 1:**
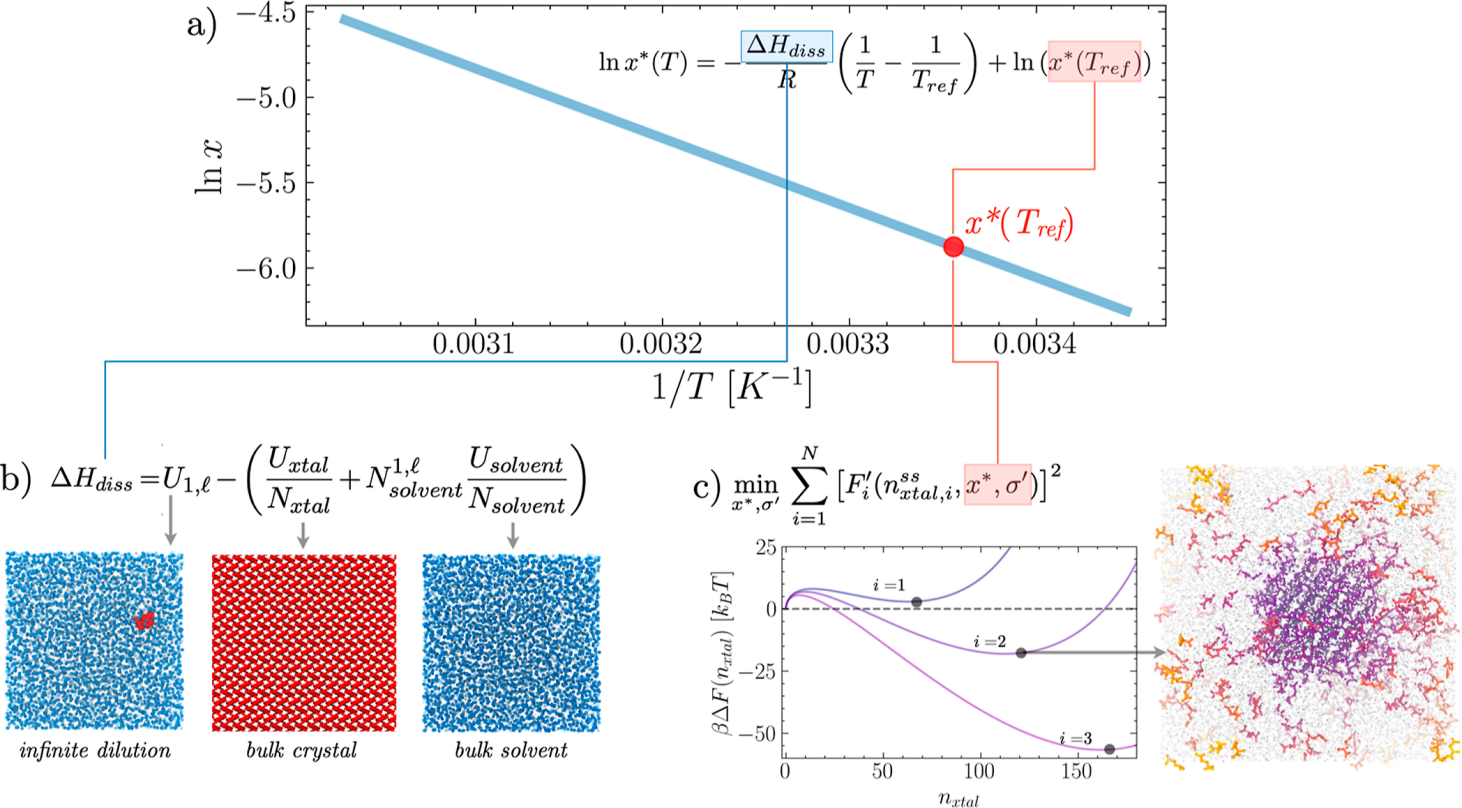
Efficiently estimating solid–liquid equilibria in solution from equilibrium MD simulations. (a) Van’t Hoff plot the solubility as a function of the temperature. (b) Simulations of the bulk solution, crystal, and solvent are used to estimate the enthalpy of dissolution Δ*H*_diss_. (c) Finite-size seeded nucleation simulations are used to independently estimate the solubility (x*) at a reference temperature. In this work, *T*_ref_ = 298.

This section introduces the theoretical background associated with the proposed analysis method. As schematically shown in Figure 1a, we aim to obtain a complete picture of the temperature-dependent, polymorph-specific thermodynamics of crystal nucleation. As such, we carried out two complementary sets of simulations. On the one hand, we perform equilibrium simulations of crystalline seeds in solution with the scope of obtaining polymorph-specific solubility and surface tension estimates at one reference temperature (*T*_ref_, highlighted indicated in red in Figure 1). On the other hand, by computing the enthalpy of dissolution from simulations of bulk solvent, solute, and solution states, we extend the information gathered at *T*_ref_ to a range of accessible temperatures.

### Thermodynamic Equilibrium for a Crystal Particle in the Isothermal–Isobaric Ensemble

The thermodynamics of nucleation of crystalline materials is typically expressed building on the foundational idea of Classical Nucleation Theory (CNT), i.e., that the free energy of a crystalline nucleus of arbitrary size *n*_xtal_ is determined by a negative, thermodynamically favorable contribution due to the difference in chemical potential between the parent, metastable, phase, and the product *stable* phase.^26^ In addition, a positive unfavorable term is determined by an interface between the parent and the product phases. These two contributions give rise to the typical free energy profile associated with the nucleation of a crystallization particle in solutions, which admits a single stationary point, a maximum, corresponding to the critical nucleus.^1,2,4,26^

When nucleation is studied in systems that deviate from the macroscopic limit due to their finite-size confinement, the thermodynamics of crystal nucleation changes, together with the shape of the free energy profile function of the crystal size.^2,27^ In the following, we shall refer to confinement and to finite-size conditions when considering a finite system at constant pressure that does not exchange matter with the surrounding environment but *can* exchange energy with a reservoir at a constant temperature. Such conditions can be practically realized in microfluidic experimental setups^28,29^ and are ubiquitous in atomistic simulations of nucleation^17,30−38^ and growth^39,40^ from the solution performed in thermostated ensembles constrained in the total number of atoms.^41^

Such a constraint implies that the total number of molecules of any species explicitly simulated is constant. Therefore, it introduces a coupling between the size of the crystal nucleus and the chemical potential of the parent phase. This leads to qualitatively and quantitatively different free energy profiles that, under these conditions, acquire a dependence on the volume of the isolated system. As discussed in detail in the literature,^2,33,35^ when confinement conditions are not too strict, the nucleation free energy profile becomes characterized by *two* stationary points. This is in contrast with the single stationary point corresponding to the critical nucleus size in macroscopic, constant composition conditions^17,26,33^ Also, in this case, one of the stationary points, the one corresponding to the smaller nucleus and associated with a maximum in Δ*F*(*n*_xtal_) defines the critical nucleus size in confined conditions. The second stationary point is a consequence of confinement at larger nucleus sizes and corresponds to a stable state realized in equilibrium conditions^17,33^

Following the derivation of ref (33), the nucleation free energy in confinement conditions, function of the number of solute molecules in the crystalline nucleus *n*_xtal_ reads
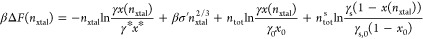
1where β = 1/*k*_B_*T*, *x*(*n*_xtal_) is the molar fraction in solution coupled to the size of the crystal nucleus *n*_xtal_, as implied by eq 2
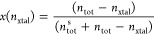
2γ, γ_0_, and γ* are the activity coefficients of the solute at molar fraction *x*(*n*_xtal_), *x*_0_ = *x*(*n*_xtal_ = 0) and at equilibrium (x*), *n*_tot_ is the total number of solute molecules in the system, *n*_tot_^s^ the total number of solvent molecules in the system, γ_s_, and γ_s,0_ the chemical potentials of the solvent at molar fraction (1 – *x*(*n*_xtal_)), and (1 – *x*_0_), respectively. Finally, σ′ is an effective surface energy term.^33^

Note that in the future we have dropped the explicit dependence on the γ term. This is because γ is dependent on the molar fraction of solute monomers in solution, and as *x* → x* the ratio of the activity coefficients γ(*x*)/γ*(x*) tends to 1. In these conditions, eq 1 depends only on the system composition and volume, i.e., *n*_tot_, *n*_tot_^s^, and on two *simulation-independent* thermodynamic parameters: the solubility x* and an effective surface energy σ′ averaged over the quasi-spherical surface of a simulated seed. Simulations performed with different setups, i.e., characterized by different values of *n*_tot_ and *n*_tot_^s^, are described by the same set of thermodynamic parameters x* and σ′. See the scheme reported in Figure 1c, where three free energies are obtained with the same x* and σ′. More precisely, for the same set of x* and σ′ parameters, a steady state crystal of size *n*_xtal_^ss^ obtained from a simulation box characterized by an assigned number of solute and solvent molecules *n*_tot_ and *n*_tot_^s^ correspond to the stable equilibrium state defined by a local minimum in eq 1. Similar to the seeding method,^13,42^ we exploit this observation by performing MD simulations initialized with a crystalline seed in the simulation box. However, while in *seeding* simulations, one is interested in finding the *unstable* critical nucleus size; here, we seed for the stable, steady-state nucleus that emerges due to finite-size effects.^17^ As discussed in the following sections, this provides a practical approach to extracting thermodynamic parameters as the steady-state cluster is reached when the system relaxes to a stable equilibrium, and therefore, its ensemble properties (including, crucially, its size) are ergodically sampled in the long-time limit.

### Estimating Solubility and Surface Tension from Equilibrium Conditions in Finite-Sized Simulations

We obtain an estimate of the parameters x* and σ′ from a set of *N* simulations at an assigned temperature *T* by noting that while each *i*th simulation, characterized by different setup conditions *n*_tot*,i*_ and *n*_tot,*i*_^s^, equilibrates to a different stable equilibrium size *n*_xtal,*i*_^ss^, the thermodynamic parameters x* and σ′ are *simulation-independent*. We indicate with *F*_*i*_^′^(*n*_xtal_, x*, σ^′^) the derivative function of βΔ*F*(*n*_xtal_) with respect of *n*_xtal_, at assigned *n*_tot*,i*_, *n*_tot,*i*_^s^, function of the parameters x* and σ′
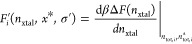
3

At equilibrium, the following condition is satisfied for every *i*th simulation performed at assigned *n*_tot*,i*_ and *n*_tot,*i*_^s^ for their equilibrium cluster size *n*_xtal,*i*_^ss^
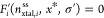
4

As such, the stationary point condition of eq 4 can be used to compute the thermodynamic parameters x* and σ′ as the solution to a nonlinear least-squares problem
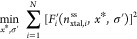
5we note that the condition established by eq 4 would be valid *also* for an unstable equilibrium state, corresponding to a local maximum of βΔ*F*(*n*_xtal_). Our approach inherently avoids such solutions as *n*_xtal,*i*_^ss^ that is estimated directly from the stable equilibrium configuration sampled by atomistic simulations at the steady state.

### Computing Solubility Curves

Given the solubility at a given reference temperature *T*_ref_, obtained by solving the problem described by eq 5, one can extend the characterization of the equilibrium thermodynamic conditions to a range of temperatures by adopting the Van’t Hoff expression obtained from applying the Gibbs-Konovalev theory of thermodynamic displacements to the solid/liquid equilibrium^10,43^
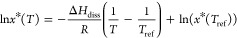
6where x*(*T*_ref_) is the solubility at a reference temperature, and Δ*H*_diss_ is the dissolution enthalpy, i.e., the enthalpy associated with transferring one mol of solute from the crystalline phase to a liquid phase, with the solvent of interest at infinite dilution. It should be noted that using eq 6 does not imply an ideal solution, but merely the fact that the activity coefficients, and Δ*H*_diss_ are constant across the temperature range investigated.^10^ Considering *T*_ref_ the temperature at which simulations are performed, x*(*T*_ref_) is obtained from the solution of the minimization problem outlined in eq 5. The dissolution enthalpy can instead be estimated by performing three additional equilibrium simulations of the bulk crystal, the pure solvent in its liquid state, and a single solute molecule in solution, approximating the infinite dilution conditions. As illustrated in Figure 1b, by defining as *U*_xtal_ the total energy of the simulated bulk crystal, *U*_solvent_ as the total energy of the pure solvent in its liquid phase, and  the total energy of the system approximating infinite dilution and containing a single solute molecule, the dissolution enthalpy can be computed as^44^
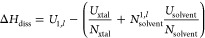
7where *N*_xtal_ is the number of solute molecules in the crystal bulk simulation, *N*_solvent_ is the number of water molecules in the pure solvent simulation, and  is the number of solvent molecules in the simulation of the solution at infinite dilution.

### Estimating Nucleation Barriers and the Limit of Kinetic Solution Stability

Combining eqs 6 and 7 allows us to obtain a model-based estimate of the solubility curve. Assuming, as often done when interpreting experimental nucleation data,^20−22^ a negligible dependence of the solid–liquid surface tension with temperature, one can obtain the classical nucleation-free energy barrier as a function of temperature and composition
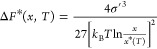
8

The limit of solution stability *x*′(*T*) is the locus of the point on the (*x*, *T*) plane that yields a nucleation barrier ΔF*(*x*, *T*) on the order of *k*_B_*T*. This corresponds to highly supersaturated conditions, where the system reaches the spinodal limit. In such conditions, the free energy barrier associated with nucleation is of the same order of magnitude as the thermal energy of the system, and nucleation ceases to be a rare event. As such, the solution becomes kinetically unstable, leading to what in experiments is often indicated with *instantaneous* precipitation. These conditions mark the upper limit of the metastable zone (as shown in Figure 4). We note that the condition ΔF*(*x*, *T*) = 3*k*_B_*T* is somewhat arbitrary and is indicative of only a range of conditions where the nucleation-free energy barrier is of the same order as the thermal energy of the system. Estimating the extent of the metastable zone completes the outline of the full phase diagram, informed only by a handful of standard MD simulations.

## Methods

### Molecular Dynamics Simulations Setup

To determine nucleation rate parameters from steady-state clusters, we initially prepared a range of differently sized seeds cut from bulk crystal. The structure files for α and β glutamic acid were obtained from the Cambridge Crystallographic Data Centre (CCDC) corresponding to deposition IDs LGLUAC01 and LGLUAC02. The primary unit cell of each polymorph was transformed into a supercell and, using Gromacs2022.4,^45^ relaxed and equilibrated for 100 ns at ambient temperature using the Berendsen thermostat. Five differently sized seeds were cut from the equilibrated bulk, where the seed diameter range ranged from 1.2 to 3.0 nm. The seeds were placed in a cubic box populated with both solute monomers and water. Glutamic acid monomers were added using Packmol^46^ at an initial guess concentration of 0.07 and 0.08 mol/kg. The water molecules were modeled using the SPC/E force field,^47^ and glutamic acid was described using the all-atom variant of the OPLS force field,^48^ with corresponding parameters assigned by the LigParGen server.^49^

Each solvated box was optimized using a steepest descent algorithm.^50^ A careful stepwise equilibration was necessary to ensure the seeds remained stable during the production runs. We, therefore, began with a 500 ps NPT equilibration, using the Bussi-Parrinello velocity rescaling thermostat,^51^ along with the Berendsen barostat^52^ with coupling constants of 0.5 and 2 ps, respectively. Periodic boundary conditions were applied in all three dimensions, and the integration time-step for the leapfrog algorithm was set to 1 fs. In all simulations, the cutoff for the van der Waals forces and electrostatic interactions was set to 1.2 nm, where a switching function was used to bring the van der Waals interactions to zero at 1.2 nm, and the long-range contributions to the electrostatic interactions were handled using the particle-mesh-Ewald method.^53,54^ The seed positions were restrained by using a harmonic potential characterized by a 1000 kJ/mol spring constant. This first step allowed the solution to equilibrate at the solution/seed interface independent of the seed dynamics. More often than not, this step resulted in some bubble formation within the solution at the box edges and is most likely due to the high tolerance threshold used with Packmol.^46^ To get rid of the bubbles quickly, we ran a short (50 ps) simulation, which compressed the systems by changing the reference pressure to 5000 bar, effectively squeezing the bubbles out of the system. Next, an annealing protocol was used to return the systems to the desired reference pressure of 1 bar. The compressed systems are annealed (at 1 bar) from 0 to 320 K in 3 ns and then gently cooled back down from 320 to 298 K in 2 ns, followed by a further 100 ps at 298 K. At this point, the bubbles were gone, and the solution was equilibrated. The position restraint on the seed was removed and placed on the monomers in solution instead. A 100 ps NPT run was carried out in which the seed and water solution were brought to equilibrium. At this point, the solution, seed, and solution-seed interface were well equilibrated, and production runs could follow. For these runs, we switched to the Parrinello–Rahman barostat^55^ and set the time step for the leapfrog algorithm to 2 fs. A single monomer located in the center of the seed was restrained to ensure that the seed remained centered within the simulation box. Each system was simulated for 300 ns at 298 K. The same protocol was followed to generate a set of stable seeds of β LGA at 290 K, used to validate the solubility prediction obtained from simulations at 298 K.

### Trajectory Analysis: Obtaining the Steady-State Nucleus Size

To track the evolution of the seeds, we need to identify which monomers in the system are classified as crystalline and which are considered to be in the liquid phase. Therefore, a descriptor that can differentiate between the two is needed. Ours is based on a distance criterion between monomers and on comparisons of relative orientations with a reference bulk crystal.^56−58^ Note that since the classification is used for an a-posteriori analysis, the description of a liquid/crystal monomer adopted here is not continuous and differentiable but binary, based on whether the cutoff criteria for distance and relative orientations are met. Thus, we define an internal vector for each monomer based on a pair of atoms (the choice is somewhat arbitrary). Then, we evaluate the polar angle between the internal vector of a given monomer and all the other analogous vectors within a radial cutoff of 6.25 and 7.0 Å for α and β, respectively (the cutoff distances are based on the first coordination shell). We can, therefore, construct a relative orientation probability density, *p*(θ), which provides a reference fingerprint of the molecular arrangements of different crystal polymorphs. Once the characteristic distributions are known, we can check which monomers form part of the seed. Thus, for a given radial cutoff distance, if the relative orientations between two monomers fall within the reference fingerprint distribution, these monomers are fed into an adjacency matrix and clustered based on a distance that is the same as the radial cutoff. The clustering algorithm, found in the MDAnalysis software package,^59^ returns the size of the largest cluster. In this way, we can track the evolution of seeds for all trajectories.

## Results and Discussion

### Solubility, Surface Tension, and Nucleation Free Energies from MD Simulations at *T*_ref_ = 298 K

We start by solving the minimization problem stated in eq 5 to estimate the solubility x*(*T*_ref_) and surface energy βσ′ from finite size simulations of β and α LGA nuclei in aqueous solution. To this aim, we perform two sets of eight and six simulations for β and α LGA, respectively, at *T*_ref_ = 298 *K*. The simulations are carried out by varying the total number of solute and solvent molecules, *n*_*i*,tot_^s^ and *n*_*i*,tot_, thus leading each simulation to converge to its steady state cluster *n*_ss,*i*_ in independent environments, i.e. we locate *n*_ss,*i*_ at different supersaturation conditions. The values of *n*_tot_^s^, *n*_tot_, and the resulting *n*_xtal_^ss^ are reported in Table 1.

**Table 1:** Simulation-Specific Parameters for β and α LGA Simulations, Including the Total Number of Solvent and Solute Molecules (*n*_tot_^s^ and *n*_tot_, Respectively), and the Observed Equilibrium Crystal Size *n*_xtal_^ss^, Observed at Steady State in Each Simulation^a^

simulation	*n*_tot_^s^	*n*_tot_	*n*_xtal_^ss^
β—*LGA*			
1	11,974	136	75
2	15,869	197	120
3	16,774	245	169
4	19,348	316	238
5	11,879	151	78
6	15,717	214	127
7	16,648	263	183
8	19,190	336	242
α—*LGA*			
1	13,769	292	114
2	15,139	360	177
3	16,841	428	242
4	13,621	310	132
5	15,038	371	176
6	16,841	428	239

a The behavior of all these simulations can be described with a single, polymorph-specific set of physical–chemical parameters, i.e., solubility (x*) and surface energy (βσ′). All simulations were performed at *T*_ref_ = 298 *K*.

We note that the equilibrium clusters of size *n*_xtal_^ss^ of the thermodynamically stable polymorph β tend to maintain a strongly ordered internal structure. Steady-state clusters of polymorph α, instead, while achieving a stable size, tend to develop a more disordered and dynamic solid/liquid interface. For both polymorphs, however, the overall cluster size stabilizes and, with it, the composition of the liquid phase. This enables using eqs 4 and 5 to characterize crystallization thermodynamics.

**Figure 2:**
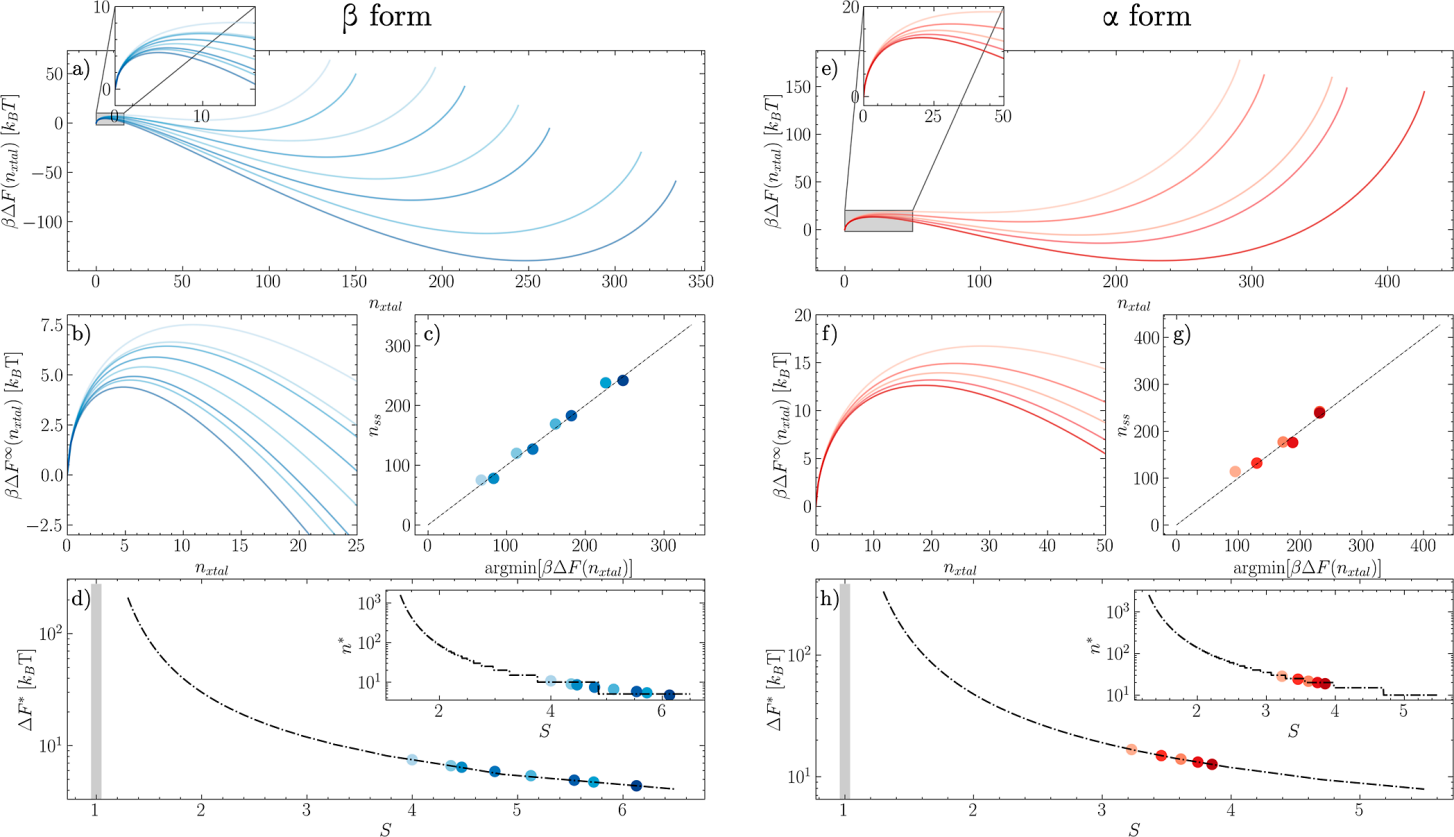
Analyzing finite-size simulations of l-glutamic acid steady-state clusters in solution in the *NPT* ensemble. On the left is an analysis of the β polymorph, and on the right is the α polymorph. (a,e) Δ*F*(*n*) obtained informing eq 2 with the best-fit results obtained solving the minimization problem outlined in eq 3. In inset a zoom on the nucleation barrier in confined conditions. (b,f) Nucleation free energy in macroscopic conditions, corresponding to the simulations analyzed in (a,e), in the vicinity of the critical Size. (c,g) Predicted steady-state condition of the free energy in confinement matches the steady-state size extracted from the simulation. (d,h) Nucleation barrier and, in the inset critical nucleus size in macroscopic conditions, as a function of supersaturation. Each shade of blue/red represents one simulation, where the same shade in each panel pertains to the same simulation.

By numerically solving the minimization problem posed by eq 5 with a nonlinear least-squares fitting algorithm, feeding the set of parameters reported in Table 1 led to the estimate of the reference solubilities *x*_β_^*^(*T* = 298) = 2.731 × 10^–3^ ± 6.680 × 10^–4^ and *x*_α_^*^(*T* = 298) = 6.460 × 10^–3^ ± 1.29 × 10^–3^ for α and β LGA, respectively. The surface energies obtained from the fitting are σ_β_^′^ = 4.810 ± 1.96*k*_B_*T* and σ_α_^′^ = 5.36 ± 1.50*k*_B_*T*. In Figure 2 panels (a,e), we report the free energy in confined conditions, Δ*F*(*n*_xtal_) obtained from eq 1 using the fitted values of solubility and surface energy reported above. Such free energy profiles exhibit significant deviations from the macroscopic behavior, most notably impacting the shape of the free energy profile in the vicinity of the critical nucleus. Such distortion can be visually appreciated by comparing the insets of Figure 2 panels (a,e), with panels (b,f). The latter report macroscopic nucleation free energy profiles βΔ*F*^*∞*^(*n*_xtal_) obtained using the fitted thermodynamic parameters x* and σ′ at supersaturation equivalent to those of the corresponding MD simulations.^17^

The self-consistency of x*(*T*_ref_) and σ′ parameters is critically assessed in Figure 2 panels (c,g), where the steady state cluster size *n*_ss_ obtained by postprocessing MD simulations are compared with the equilibrium cluster size theoretically predicted using the fitted parameters x*(*T*_ref_) and σ′. Notably, for both α and β LGA models investigated in this work, we obtain an excellent agreement, thus verifying that the behavior of all simulations of a given polymorph can be rationalized on the basis of a single set of thermodynamic parameters, as expected.^17^

Given the internal consistency of the model free energy profile obtained, we use it to estimate the nucleation barrier and critical nucleus sizes of the α and β LGA across supersaturation conditions. The results obtained are reported in Figure 2 panels (d,h).

**Figure 3:**
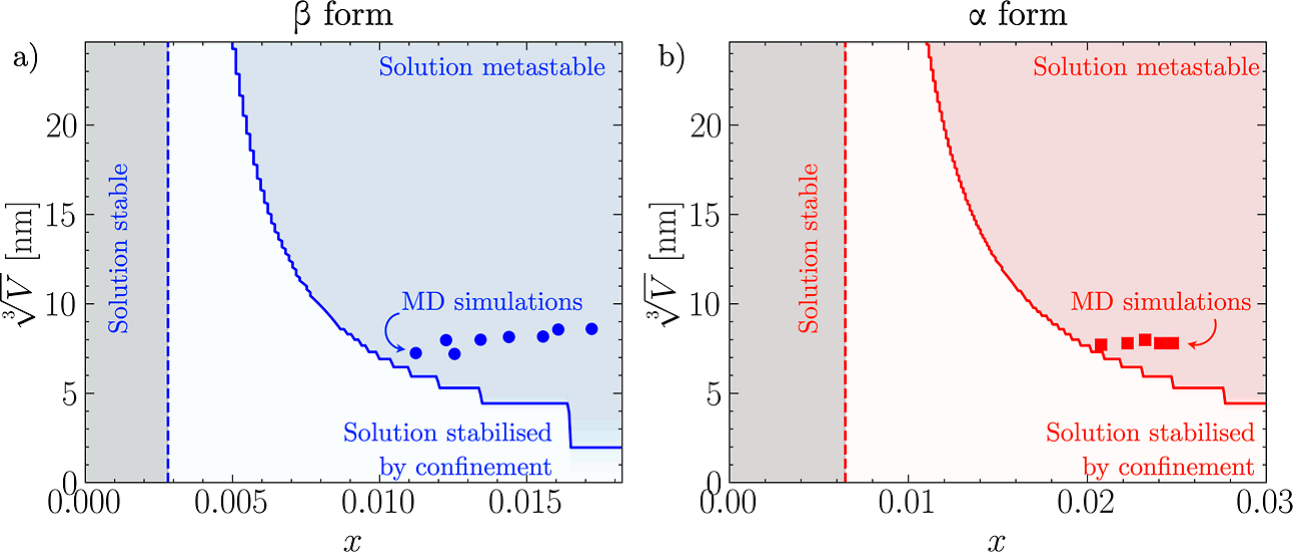
Aqueous LGA solutions stability maps in confinement. (a) Stability map with respect to β-LGA crystals. (b) Stability map with respect to α LGA crystals. The data sets illustrated with solid markers represent conditions corresponding to the MD simulations analyzed in this work. The white domain represents conditions where a supersaturated solution is stabilized by confinement. The domain in color corresponds to conditions where confinement does not inhibit nucleation.

Finally, we extend our understanding of confinement effects in simulations by mapping the thermodynamic stability in the confinement of aqueous LGA solutions against either the α and β LGA polymorphs at *T*_ref_. The solution stability maps under finite-size conditions identify three types of subdomains in the composition/volume plane. The first, represented in gray in Figure 3a,b corresponds to conditions where a solution would be thermodynamically stable in either confined or macroscopic conditions (i.e., the solution is undersaturated). The second, represented in white in both panels (a,b), represents conditions where confinement induces the stabilization of nominally supersaturated solutions, which in macroscopic conditions eventually nucleate and grow crystals. The third domain, represented in color, corresponds to conditions in which the free energy surface under confinement admits a local minimum corresponding to a cluster in equilibrium with a confined solution. In both cases, the MD-simulated conditions are consistently classified in this domain for both polymorphs.

### Solubility Curves and Limit of Solution Stability

**Figure 4:**
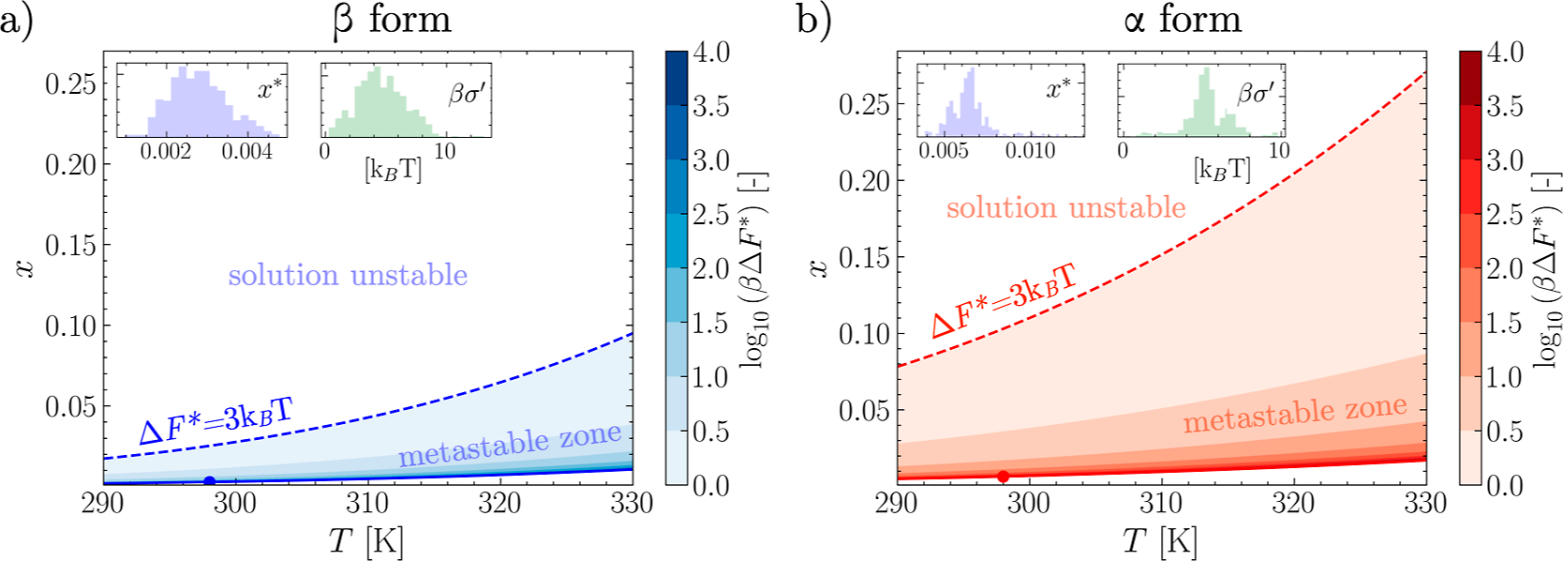
MD-informed solubility curves and limit of solution stability allow to computationally identify the boundaries of the metastable zone for both the β (a) and α (b) polymorphs of LGA. The log of the nucleation barrier is mapped within the metastable zone and represented as a contour map. The solubility at *T*_ref_ = 298 *K* is reported as a circle. In inset to both (a,b), the distributions of the parameters x* and β obtained from the bootstrap analysis used to estimate the parameters confidence intervals.

Starting from solubility and surface energy computed at *T*_ref_, we employ eq 6 to estimate the entire solubility curve across temperatures. To this aim, we compute the dissolution enthalpy, as expressed in eq 7. We perform simulations for the bulk phase of α and β LGA polymorphs modeling large crystal supercells containing *N*_α_ = 4800 and *N*_β_ = 5376 glutamic acid molecules. A simulation of  water molecules was employed to compute the potential energy for pure water, while a single LGA molecule was dissolved in the same box to estimate the potential energy at infinite dilution. This led to estimates of Δ*H*_diss_^β^ = 33.9 ± 13.5 and Δ*H*_diss_^α^ = 24.7 ± 13.5 kJ mol^–1^. The error associated with the two estimates is the same as it is dominated by the terms  and , which are common to both cases. As shown in Figure 4, this allows us to estimate the location of the solubility curve in the (*x*, *T*) plane for both α and β, as expressed in eqs 9 and 10
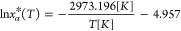
9
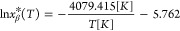
10

The solubility curves are represented as solid lines in Figure 4a,b, where the solubility at *T*_ref_ is shown as a solid circle. With x*(*T*) known, one can estimate the barrier to nucleation ΔF* across temperatures and composition by applying eq 8. The barrier, mapped with colored isocontours in Figure 4a,b, diverges for conditions approaching the solubility line and vanishes moving away from such a line. To qualitatively estimate how the nucleation barrier vanishes for different polymorphs and thus provide a computational *upper* bound to the metastable zone, we locate the limit of solution stability as the locus of points where ΔF* = 3*k*_B_*T*, as discussed in the Methods section. Such limits are represented with dashed lines in Figure 4a,b.

### Comparison with Experiments

**Figure 5:**
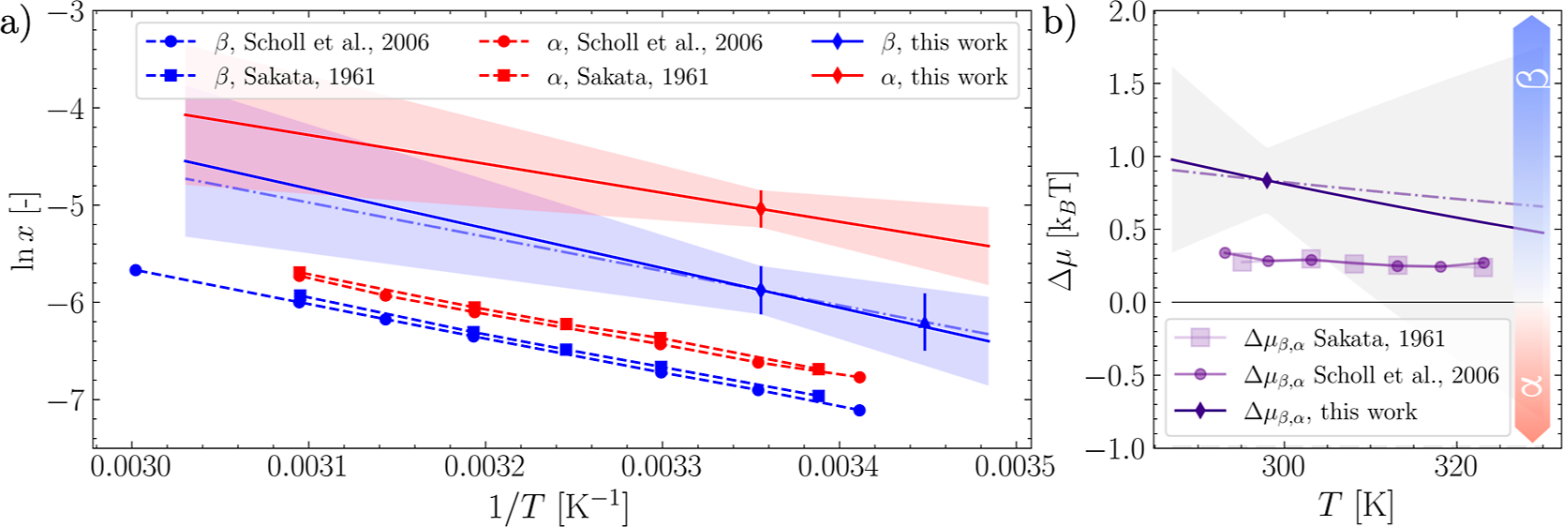
(a) Comparing the solubility curves of α and β glutamic acid obtained from MD data with experimental solubility curves obtained by Schöll et al.^20^ and Sakata.^18^ The temperature dependence, as well as the relative solubility of the two polymorphs, are well captured by simulations. However, the computationally predicted solubility is roughly twice as large as the experimental one across both polymorphs. The data point represented as a triangle is the result of an independent set of equilibrium seed simulations at *T* = 290 K. (b) Simulations and experiments consistently identify β as the most stable polymorph, leading to a monotropic phase diagram and estimate as < than *k*_B_*T* the chemical potentials difference between the two polymorphs across the whole experimentally accessible range of temperatures. In both panels, the dash-dot line represents an estimate obtained from two sets of simulations at different *T*, while the solid line represents results obtained from calculations at *T* = 298 K.

The MD-based estimates of the solubility curves obtained from α and β LGA are compared to their experimental counterparts in Figure 5. In panel (a), it can be seen that while the absolute value of solubility predicted by atomistic MD simulations is larger than its experimentally measured counterpart, the temperature scaling, i.e., the slope of the ln x*(*T*) lines is close to that of the experimental data. The overestimation of the solubility by a factor of 2 is consistent with an error of the order of *k*_B_*T* in the dissolution free energy, which is well within the typical accuracy of classical force fields. In fact, errors of this magnitude are common in many well-studied systems, such as NaCl in water, modeled with the Joung-Chetam and SPC force fields. The value of Δ*H*_diss_ obtained from simulations and the corresponding quantity obtained from experiments are instead in good agreement. For instance, a linear fit of the experimental solubility curves for the α and β polymorphs yields Δ*H*_diss_^α,exp^ = 27.19 kJ/mol (Δ*H*_diss_^α,MD^ = 24.7 ± 13.5 kJ/mol), and Δ*H*_diss_^β,exp^ = 29.02 kJ/mol (Δ*H*_diss_^β,MD^ = 33.9 ± 13.5 kJ/mol). To further validate the estimate of the solubility curve from a single *x*(*T*_ref_), we explicitly performed simulations of equilibrated seeds of the β form at *T* = 290 K, obtaining a solubility of x*(290 *K*) = 0.002 ± 6 × 10^–4^, in excellent agreement with the prediction obtained from simulations at 298 K as shown in Figure 5a. Obtaining solubilities at two different temperatures enables an independent estimate of the Δ*H*_diss_ yielding a value of 29.3 kJ mol^–1^, well within the error bar of the estimate obtained from eq 7 and remarkably close to the experimental value of 29.02 kJ mol^–1^. As a consequence, also the solubility curve computed from two independent estimates of solubility (reported as a dashed-dotted line in Figure Figure 5a) results in excellent agreement with the estimate obtained from the single-temperature prediction (solid line). Finally, modeling results predict that the β polymorph is the most stable across the entire range of experimentally accessible temperatures, consistent with experimental observations. The difference in thermodynamic stability between polymorphs can be quantitatively assessed as a function of temperature, as in eq 11
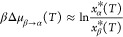
11

In Figure 5b, we report βΔμ_β→α_(*T*) computed from the experimental data sets of refs (18 and 20), together with an MD estimate of the same quantity obtained from our computationally predicted solubility curves. The solid line represents a single temperature estimate. In contrast, the dashed dot line represents the estimates where the contribution of the β polymorph is estimated from the independent values of solubility at *T* = 290 and 298 K. This result clearly shows how the slight difference in the slope of the solubility curve of the β form has a remarkable impact on the estimate of the relative stability of the two polymorphs. Consistent with experiments, βΔμ_β→α_(*T*) is positive, underscoring the fact that β is the stable form. Remarkably, βΔμ_β→α_(*T*) estimated from experimental data is of the order of 0.5 *k*_B_*T*, underscoring the extremely small difference in free energy between competing polymorphs. This observation, coupled with the sensitivity of the slope of βΔμ_β→α_ on slight variations in the free energetics of dissolution of the two polymorphs, underscores how, even if the MD-based prediction of the temperature-dependent thermodynamic stability is in good qualitative agreement with experiments, subtle differences in the energetics can lead to sizable discrepancies in the prediction of the experimental observables and significant uncertainties in the location of key features of the polymorphic phase diagram, such as crossover temperatures.^7^

## Conclusions

In this work, we demonstrate how combining equilibrium MD simulations with a theoretical understanding of nucleation free energy profiles in confined systems can efficiently produce a complete description of crystal-solution thermodynamics from first principles. We apply our method to α and β LGA crystallizing in aqueous solutions, showing that an off-the-shelf combination of force fields (OPLS solute, SPC water) leads to a surprisingly accurate description of the energetics of dissolution and the relative thermodynamic stability between polymorphs. Nevertheless, the exponential relationship between the chemical potential and solubility limits the quantitative agreement of the solubility curves. For instance, errors of the order of *k*_B_*T*, in both the enthalpy of dissolution Δ*H*_diss_, and in the Δμ_β→α_, are associated with solubility estimates roughly twice the experimental solubility across the entire range of temperatures investigated. This observation is not unusual, as vastly adopted molecular models, for which a phase diagram has been studied in detail, suffer similar limitations in accuracy^60^ and property prediction; data-driven algorithms aimed at predicting solubility display a similar level of accuracy. A critical outcome of the approach we propose in this work is that it provides an efficient route to estimate emergent properties at finite temperatures for multicomponent systems that would otherwise involve algorithmically sophisticated and computationally expensive simulations. Moreover, when applied to characterize finite-size simulation, it can efficiently identify regions of volume and composition space where nucleation is attainable and it becomes feasible to deploy unseeded, enhanced sampling methods to investigate nucleation mechanisms. Combining these outcomes is key to efficiently developing more realistic molecular models and effectively deploying simulations in large-scale computational screenings of polymorphic stability.

## Supporting Information Available

Probability distribution of relative orientation of glutamic acid molecules in the α and β polymorphs used to define criteria to estimate the size of the steady-state cluster in simulations (PDF)
