# Supplementary material for: Solution Thermodynamics of l-Glutamic Acid Polymorphs from Finite-Sized Molecular Dynamics Simulations
Source: Ind Eng Chem Res. 2025 Jan 7;64(2):1309–18. doi: 10.1021/acs.iecr.4c02558 (PMC11741101; doi:10.1021/acs.iecr.4c02558)
Supplement: Supplementary file 1 — ie4c02558_si_001.pdf [file ie4c02558_si_001.pdf]

Supplementary Information: Solution Thermodynamics of L-Glutamic Acid Polymorphs from Finite-Sized Molecular Dynamics Simulations

Fabienne Bachtiger, Aliff Rahimee, Lunna Li, and Matteo Salvalaglio*

Thomas Young Centre and Department of Chemical Engineering, University College London, London WC1E 7JE, UK

E-mail: m.salvalaglio@ucl.ac.uk

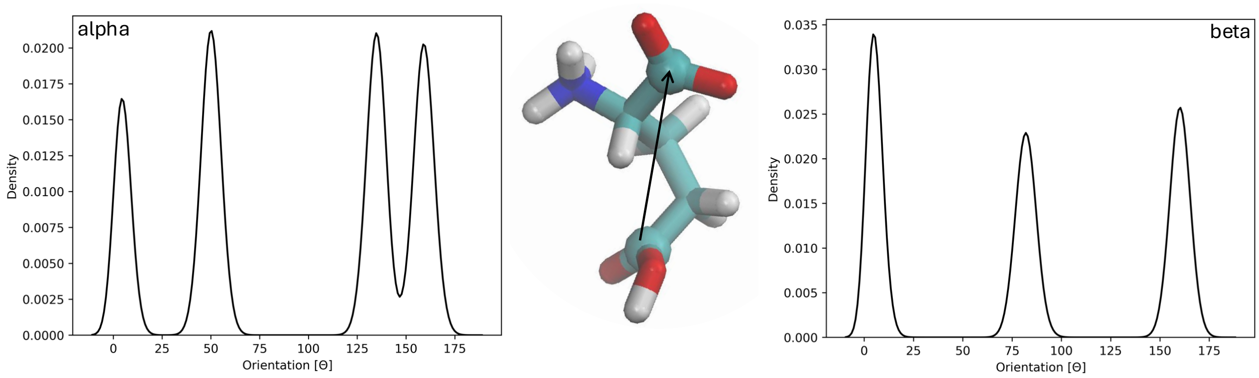

Figure S1: Probability distribution of relative orientations. Each glutamic acid monomer within a crystalline bulk is transformed into a vector. The relative orientation of each vector and its neighbors (for a given radial cut-off) is computed from which a characteristic fingerprint can be found. Hence, the orientations of glutamic acid monomers within a solid phase can be distinguished by those found in a solution by assuming a distance criterion between monomers and relative orientations falling into one of the peaks in the distributions, either for α or β, respectively.
